# *Arabidopsis thaliana GYRB3* Does Not Encode a DNA Gyrase Subunit

**DOI:** 10.1371/journal.pone.0009899

**Published:** 2010-03-26

**Authors:** Katherine M. Evans-Roberts, Christian Breuer, Melisa K. Wall, Keiko Sugimoto-Shirasu, Anthony Maxwell

**Affiliations:** 1 Department of Biological Chemistry, John Innes Centre, Norwich, United Kingdom; 2 RIKEN Plant Science Center, Yokohama, Kanagawa, Japan; University Paris 7, France

## Abstract

**Background:**

DNA topoisomerases are enzymes that control the topology of DNA in all cells. DNA gyrase is unique among the topoisomerases in that it is the only enzyme that can actively supercoil DNA using the free energy of ATP hydrolysis. Until recently gyrase was thought to be unique to bacteria, but has now been discovered in plants. The genome of the model plant, *Arabidopsis thaliana*, is predicted to encode four gyrase subunits: AtGyrA, AtGyrB1, AtGyrB2 and AtGyrB3.

**Methodology/Principal Findings:**

We found, contrary to previous data, that AtGyrB3 is not essential to the survival of *A. thaliana*. Bioinformatic analysis suggests AtGyrB3 is considerably shorter than other gyrase B subunits, lacking part of the ATPase domain and other key motifs found in all type II topoisomerases; but it does contain a putative DNA-binding domain. Partially purified AtGyrB3 cannot bind *E. coli* GyrA or support supercoiling. AtGyrB3 cannot complement an *E. coli gyrB* temperature-sensitive strain, whereas AtGyrB2 can. Yeast two-hybrid analysis suggests that AtGyrB3 cannot bind to AtGyrA or form a dimer.

**Conclusions/Significance:**

These data strongly suggest that AtGyrB3 is not a gyrase subunit but has another unknown function. One possibility is that it is a nuclear protein with a role in meiosis in pollen.

## Introduction

DNA topoisomerases are essential enzymes that control the topology of DNA [1], [2]. Topoisomerases are classified into two types, I and II, depending on whether they catalyse reactions involving the transient breakage of one or both strands of DNA [3]. DNA gyrase is a type II topoisomerase, and is the only enzyme able to introduce negative supercoils into DNA; supercoiling is achieved at the expense of ATP hydrolysis [4], [5]. DNA gyrase consists of two subunits, GyrA and GyrB in bacteria, which are ∼100 kDa in MW (97 kDa and 90 kDa in *Escherichia coli*). Through structural and mechanistic studies a picture has emerged as to how gyrase introduces supercoils into DNA [3], [4], [5]. The enzyme binds a DNA segment of ∼130 bp, which is wrapped around the A_2_B_2_ complex. This segment is cleaved in both strands with the formation of phosphotyrosine bonds between the broken DNA and the GyrA subunit. The binding and hydrolysis of ATP, by the GyrB subunit, facilitates the passage of another part of the wrapped segment through the break. This process reduces the linking number of the DNA by two, i.e. two negative supercoils are introduced.

Until comparatively recently gyrase was thought to be unique to bacteria and this, plus the formation of enzyme-stabilised double-stranded breaks in DNA, has made gyrase an attractive target for antibacterial agents [6], [7]. In 2004 it was shown that the plants *Arabidopsis thaliana* (At) and *Nicotiana benthamiana* also contain DNA gyrase [8], [9]; the presence of gyrase in the monocot rice (*Oryza sativa* [10]) suggests that the enzyme is present throughout the plant kingdom. Gyrase coexists with DNA topoisomerase II [11], which is a nuclear enzyme present in all eukaryotes. Within the *A. thaliana* genome there is one *GYRA* gene (At3g10690) and three *GYRB* genes (At3g10270, At5g04130 and At5g04110) [11]. *GYRA*, *GYRB1* and *GYRB2* all encode transit peptides, which target them to chloroplasts and mitochondria [9]. However, AtGyrB3 does not contain a transit peptide, and the sub-cellular localisation of the protein is not known. AtGyrB3 has been reported to be essential as a T-DNA insertion in the *GYRB3* gene (At5g04110) is reported to be seedling-lethal and to complement a temperature-sensitive *gyrB* mutation in the *E. coli* strain N4177 [9], implying that it is a component of an active gyrase in *A. thaliana*. However, at 61 kDa, AtGyrB3 is significantly smaller than AtGyrB1, AtGyrB2, or other bacterial GyrBs and is missing a significant portion of the N-terminus, including part of the ATPase domain, when compared to other GyrB proteins. Phylogenetic analysis of Arabidopsis gyrases revealed that *GYRA*, *GYRB1* and *GYRB2* align with the gyrases of other plants and the endosymbiotic bacteria, whereas *GYRB3* aligns more closely with the eukaryotic topoisomerase IIs [9]. Given the uncertainties surrounding AtGyrB3, we have investigated it further with the aim of establishing whether it is a genuine gyrase B subunit.

## Results

### Phenotypic analysis of *AtGYRB3* knockout mutants

Previous work had suggested that an insertion into the *A. thaliana GYRB3* gene (T-DNA line SAIL_390_D05) led to a seedling-lethal phenotype [9]. We have now repeated this analysis using three independent insertion lines, at least one of which has an insertion into the coding sequence of the gene (Figure S1), and found no evidence of any obvious phenotypic differences from wild-type plants (data not shown). We therefore conclude that *GYRB3* is not an essential gene in *A. thaliana* and that the previous result was incorrect [9] (in this case, only a single insertion line was examined).

### AtGyrB3 lacks key motifs of the DNA gyrase B protein

It had previously been noted that the putative AtGyrB3 protein was significantly shorter at its N-terminal end than the other two *A. thaliana* GyrBs [9]. To further investigate this, we performed 5′-RACE analysis and found that the 5′-end of the gene had indeed been correctly annotated (data not shown). We also carried out 3′-RACE analysis and confirmed that the 3′-end was also annotated correctly (data not shown). In addition, organellar-targeting experiments had shown that whereas AtGyrA, AtGyrB1 and AtGyrB2 were targeted to mitochondria and/or chloroplasts, AtGyrB3 protein did not appear to be organellar-targeted, presenting the puzzle of a putative GyrB with no GyrA counterpart [9]. Indeed analysis using MultiLoc [12] suggests that GyrB3 may be nuclear-targeted.

The protein sequences of *E. coli* GyrB, AtGyrB1, AtGyrB2 and AtGyrB3 were aligned using the Match-Box server [13]. AtGyrB3 is missing the first ∼200 amino acids when compared to *E. coli* GyrB (Figure S2). This corresponds to the N-terminal sub-domain of the ATPase region, which forms the majority of the interactions with bound ATP and adopts a GHKL fold [14], and is essential for DNA gyrase activity. Seven conserved motifs found in all type II topoisomerases (RPXXYIGS, GXGXP, GGXXGXG, EGDSA, PL(R/K)GK(I/L/M)LNV, IM(T/A)D(Q/A)DXD and YKGLG) [15], [16] are found in AtGyrB1 and AtGyrB2, but not in AtGyrB3. Bacterial GyrB contains four conserved domains, which are all present in AtGyrB1 and AtGyrB2, these are the ATPase domain, the transducer domain, the toprim domain and the C-terminal domain or tail [17]; note that *E. coli* GyrB has a ∼170 amino acid insertion before the tail domain. However analysis of the AtGyrB3 sequence reveals that it only contains the transducer domain, which is the second (C-terminal) domain of the ATPase region, consisting of a four-stranded β-sheet backed by two α-helices [18], and is essential for ATP hydrolysis. An alignment of the AtGyrB3 transducer domain and other plant and bacterial transducer domains was performed using the Match-Box server [13]; the AtGyrB3 sequence clearly aligns with the transducer sequences from known DNA gyrases (Figure S2B).

One point of interest is that AtGyrB3 has a glutamine at position 157, which aligns with Lys337 in the *E. coli* GyrB protein; this lysine appears to be conserved across plant species. The majority of the amino acids in GyrB that interact with bound ATP are found in the N-terminal region (residues 2-220 in *E. coli*) [18], however there are two amino acids from the transducer domain that also interact with ATP: Gln335 and Lys337. Lys337 binds to the γ-phosphate of ATP, and has been shown to have a critical role in the ATPase reaction of GyrB [19]. *E. coli* GyrB with a Lys337Gln mutation shows no detectable ATPase or supercoiling activity, which again implies that AtGyrB3 is unlikely to be an ATPase.

**Figure 1:**
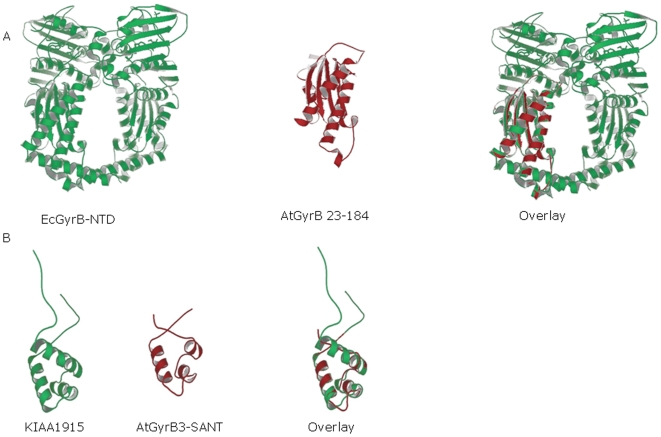
Structure predictions. A. The predicted structure of AtGYRB3 (amino acids 23 to 184). From left to right: the structure of the dimer of the 43 kDa domain of GyrB from *E. coli* with ADPNP [18]; the structure predicted for the AtGYRB3 transducer domain; the two structures merged together. B. Prediction of the structure of the AtGYRB3 SANT domain. From left to right: the solution structure of the SANT domain from the human myb-like protein KIAA1915 (Yoneyama et al, unpublished data); the predicted structure of the AtGYRB3 SANT domain; both structures superimposed.

The sequence of AtGyrB3 from amino acids 23 to 184 has close similarity to that of the *E. coli* GyrB transducer domain. The SWISS-MODEL server [20] was used to predict the structure of AtGyrB3 using homology modelling, with the structure of the 43 kDa N-terminal domain of *E. coli* GyrB as a template (Figure 1A); it must be emphasised that this is merely a hypothetical structure. The sequence of AtGyrB3 from amino acids 185 to 465 was found to have no significant matches with protein sequences in the available databases. The Eukaryotic Linear Motif server [21] predicts that AtGyrB3 has a SANT domain between amino acids 466 and 519. The SANT (SWI3, ADA2, N-CoR and TFIIIB B″) domain is a DNA-binding domain that is similar to those found in MYB-related proteins [22]. The MYB DNA-binding domain consists of up to three imperfect repeats, each forming a helix-loop-helix structure. A further characteristic of the MYB repeat is three regularly spaced tryptophans. In the SANT domain the second and third tryptophans are often replaced by other aromatic residues. The predicted SANT domain of AtGyrB3 was aligned with the SANT domains of *Saccharomyces cerevisiae* SWI3, *S. cerevisiae* ADA2, mouse N-CoR and *S. cerevisiae* TFIIIB B″ (Figure S2C). The alignment revealed that *AtGYRB3* contains two of the conserved tryptophans found in MYB and SANT domains, and a tyrosine in place of the third. SWISS-MODEL [20] was used to predict the structure of the SANT-like domain of *AtGYRB3*, based on its sequence similarity with the SANT domain of the human myb-like protein KIAA1915, the structure of which has been solved (Yoneyama et al, unpublished data [23]). This region of *AtGYRB3* is predicted to form three α-helices, resembling the MYB domain helix-loop-helix structure (Figure 1B).

Taken together these analyses suggest that AtGyrB3 is not a gyrase subunit, is unlikely to have an ATPase domain, but may have a SANT domain, suggesting that it might be a DNA-binding protein.

### The expression of *AtGYRB3* does not mirror that of other gyrase genes

It is possible to monitor Arabidopsis gene expression across the genome using microarrays. In the Affymetrix ATH1 array, oligonucleotide probes representing approximately 23,750 genes have been synthesised on glass slides [24]. Thousands of experiments have been performed using this array, and many of these are publicly available. The GENEVESTIGATOR database is a tool for studying more than 1800 of these experiments to look for patterns of gene expression [25], [26]. We used this database to analyse the expression levels of the Arabidopsis gyrase genes in selected experiments to see whether *GYRB3* expression correlated with that of the other gyrase genes. We selected experiments where the expression levels of the gyrase genes are most likely to vary and so examined gene expression throughout the cell cycle and during the growth of a cell culture.

**Figure 2:**
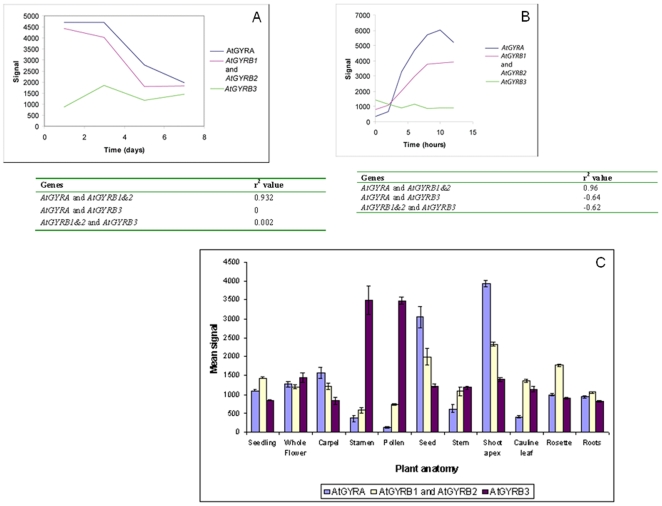
Gene expression analysis. A: Gene expression in *Arabidopsis* cell culture following sub-culture into fresh media [27]. Day 1 = late lag/early exponential phase; day 3 = mid-exponential phase; day 5 = transition from exponential phase to stationary phase; day 7 = stationary phase. Pearson's correlation coefficients are given in the table; where r^2^ = 1 is a positive correlation and r^2^ = 0 is no correlation. B: Gene expression in an *Arabidopsis* cell culture after synchrony in G1 phase [27]. G1 phase = 0 to 6 h; S phase = 6 to 11 h; G2 = 11 to 12 h. Correlation coefficients are given in the table. C: Expression of *GYRA*, *GYRB1*, *GYRB2* and *GYRB3* in different parts of the *Arabidopsis* plant [26]. *GYRB3* is most strongly expressed in the stamens and pollen, whereas *GYRA*, *GYRB1* and *GYRB2* are most strongly expressed in the seed and shoot apex.

The cell culture microarray experiment used the Arabidopsis cell line MM2d, which was sub-cultured into fresh media in order to synchronise the cells [27], [28]. Samples were harvested at two-day intervals, until stationary phase was reached. We used GENEVESTIGATOR to extract the signal values for the Arabidopsis gyrase genes for this experiment, and these are displayed in Figure 2A. As *GYRB1* and *GYRB2* have very similar sequences, the same oligonucleotide probe binds to both of them and they cannot be distinguished. Both *GYRA* and *GYRB1&2* have their highest expression levels at the late lag to mid-exponential phases of growth, with decreasing expression later in the exponential phase and into the stationary phase. *GYRB3* expression is lower and does not vary much in this time course (Figure 2A). Pearson's correlation coefficients are given in Figure 2A; there is a correlation between *GYRA* and *GYRB1&2*, with an r^2^ value of 0.932. However there is not a correlation between *GYRB3* and any other gene. We also looked at the expression of the gyrase genes from an experiment in which synchronised MM2d cells were allowed to progress through the cell cycle [27]. The cell culture was starved of sucrose, resulting in an accumulation of cells in the G1 phase of the cell cycle. The re-addition of sucrose then allows cells to progress through the cell cycle synchronously [28]. Samples were taken at two-hourly intervals, and RNA was extracted and hybridised to an ATH1 chip. The expression levels for the gyrase genes are presented in Figure 2B. *GYRA* expression increases throughout G1 and S phase, before decreasing in G2 phase. *GYRB1&2* expression also increases in G1 and S phase, but levels out during S phase and into G2 phase. *GYRB3* expression does not vary much in this experiment. The correlation coefficients in Figure 2B confirm that there is a correlation between the expression levels of *GYRA* and *GYRB1&2*, but not between *GYRB3* and either *GYRA* or *GYRB1&2*.

The Gene Atlas tool in GENEVESTIGATOR uses the signal values for a particular gene across many experiments to determine a mean signal value for particular plant organs or tissues [26]. We used this tool for each of the Arabidopsis gyrase genes to see in which parts of the plant they were particularly strongly expressed. The results for selected organs and tissues are shown in Figure 2C. One would expect the gyrase genes to be most strongly expressed in areas where there is a high rate of organellar replication. *GYRA* and *GYRB1&2* show similar expression patterns, being most strongly expressed in the seed and shoot apex. However, *GYRB3* is expressed most strongly in the stamens and pollen, while both *GYRA* and At*GYRB1&2* have relatively low expression in these tissues.

The lack of correlation in expression between *GYRB3* and the other gyrase genes further supports the contention that AtGyrB3 is not a gyrase subunit.

### *GYRB3* does not complement an *E. coli gyrB* temperature-sensitive strain

*A. thaliana GYRB3* has been reported to complement a temperature-sensitive (ts) *gyrB* mutation in the *E. coli* strain N4177 [9]. However, this result is very surprising as *GYRB3* is missing part of the ATPase domain of GyrB, and several conserved residues. As these earlier experiments were carried out in vectors where complementation relied on leaky expression, we decided to repeat these experiments using more appropriate plasmids.

Plasmid pJB11 contains *E. coli gyrB* under the control of its own promoter [29]; this plasmid successfully complements the ts strain (Table 1). We therefore constructed a series of plasmids, based on pJB11, containing *A. thaliana GYRB* genes. *E. coli* strain N4177 was transformed with plasmids pKER15 (vector), pKER18 (*GYRB3*), pKER19 (*E. coli gyrB*) and pKER22 (*GYRB2*) and grown at 30°C and 43°C (Table 1). *E. coli* strain MM28, which is closely related to N4177 but does not contain the ts mutation, was also used and grown at 30°C and 43°C, in order to show that *E. coli* can grow at both temperatures. The experiment revealed that *GYRB3* is unable to complement the ts strain, whereas *GYRB2* and *E. coli gyrB* can, suggesting that *GYRB2* encodes a functional gyrase B protein but that *GYRB3* does not.

**Table 1:** AtGyrB3 does not complement an *E. coli gyrB^ts^* strain.

		Growth^a^	
*E. coli* strain	Plasmid	30°C	43°C
MM28	None	+++	+++
N4177	None	+++	-
N4177	pKER15 (vector)	+++	-
N4177	pJB11 (*EcgyrB*)	+++	+++
N4177	pKER19 (*EcgyrB*)	+++	++
N4177	pKER18 (*AtGYRB2*)	+++	++
N4177	pKER22 (*AtGYRB3*)	+++	-

a + indicates growth;

- indicates no growth.

### AtGyrB3 does not support DNA supercoiling

*AtGYRB3* was cloned into the expression vectors pET17b and pET19b; both these clones over-express AtGyrB3 under the control of the T7 promoter, but pET19b adds an N-terminal His-tag. The plasmids were first transformed into BL21 (DE3) pLysS and tested in trial inductions, and were found to successfully over-express AtGyrB3 (data not shown). AtGyrB3 expression was tried in various *E. coli* strains, with inductions at different temperatures, but in all cases only insoluble protein was produced. In the case of protein expressed in *E. coli* Rosetta pLysS cells, the insoluble (untagged) protein was subjected to refolding. The soluble refolded protein fraction was partially purified and confirmed as AtGyrB3 by mass spectrometry (data not shown).

The purified protein was assayed for supercoiling on its own and in the presence of *E. coli* GyrA; AtGyrA is not available due to difficulties in preparing this protein [9]. We found no evidence of any supercoiling activity (data not shown). It should be noted that in previous work we showed supercoiling activity with AtGyrB2 in the presence of *E. coli* GyrA [9].

A GyrA-affinity column [30] was used to test for interaction between AtGyrB3 and *E. coli* GyrA. The GyrA affinity column was first tested with *E. coli* GyrB, which bound and eluted as expected. In the case of AtGyrB3 we found no evidence for it binding to *E. coli* GyrA, again supporting the idea that it is not a gyrase protein (data not shown).

### Yeast two-hybrid analysis of *A. thaliana* gyrase proteins

In order to ascertain whether AtGyrB3 could interact with AtGyrA, we performed yeast two-hybrid assays. The results in Table 2 suggest that AtGyrB1 interacts with AtGyrA, but that neither AtGyrB2 nor AtGyrB3 show evidence of interaction. In addition, we found no evidence of interaction of AtGyrB3 with itself. As a positive control we included Bin4, a component of the plant DNA topoisomerase VI complex [31], that is known to interact with *A. thaliana* TFII (Transcription Factor II) in yeast (CB, unpublished data). These results were corroborated by a yeast two-hybrid service (Hybrigenics) who found, using AtGyrA as the bait, AtGyrB1 and a transcription factor AtMYB124 as interactors, but no evidence of interaction between AtGyrA and either AtGyrB2 or AtGyrB3. When AtGyrB3 was used as the bait, evidence for interaction with a number of proteins was found, including ethylene-inducing calmodulin-binding protein (At5g09410), an oxidoreductase (At1g16720) and ubiquitin-protein ligase (At1g67530), but none of the other gyrase subunits.

**Table 2:** Yeast two-hybrid analysis of *A. thaliana* gyrase proteins.

AD fusion^a^						
		None	Bin4	AtGyrB1	AtGyrB2	AtGyrB3
	None	-	NT	-	-	-
	TFII	NT	+	NT	NT	NT
	AtGyrA	-	NT	+	-	-
	AtGyrB3	-	NT	NT	NT	-

a AD fusion = fusion with the activation domain of the *E. coli* peptide B42; BD fusion = fusion with the DNA-binding domain of LexA; TFII = Transcription Factor II;

+ indicates interaction;

- indicates no interaction; NT indicates not tested

## Discussion

The Arabidopsis genome encodes three putative GyrB subunits: AtGyrB1, AtGyrB2 and AtGyrB3 [11]. AtGyrB1 and AtGyrB2 are very similar, and align with bacterial GyrB proteins with the addition of N-terminal transit peptides targeting them to the endosymbiotic organelles [9]. AtGyrB3 appears to be missing a significant portion of the GyrB N-terminal domain, including residues essential for ATP hydrolysis. We have shown that the 5′ end of *AtGYRB3* is correct by 5′ RACE, confirming that these residues are likely to be missing. Sequence analysis revealed that even in regions with significant homology to other GyrB proteins, AtGyrB3 is missing key conserved motifs found in all type II topoisomerases (Figure S2). Homology modelling of AtGyrB3 based on the structure of the 43 kDa N-terminal domain of *E. coli* GyrB [18] predicted that part of AtGyrB3 forms a structure corresponding to the “wall” of the GyrB cavity (Figure 1A). The C-terminal end of AtGyrB3 has homology to the SANT DNA-binding domain. Homology modelling based on the SANT domain of the human protein KIAA1915 [23] predicts that this region of AtGyrB3 forms three short α-helices separated by two loops (Figure 1B). This structure has been shown to bind DNA in MYB proteins [32]. A BLAST search performed with AtGyrB3 reveals no similar proteins. Therefore *AtGYRB3* may have evolved in Arabidopsis from the fusion of a gene encoding the gyrase transducer domain and one encoding a DNA-binding domain from a transcription factor.

We over-expressed AtGyrB3 in *E. coli* but were unable to express soluble protein; insoluble protein was denatured in urea and refolded. This resulted in some soluble protein that was partially purified. This protein was unable to supercoil relaxed DNA when combined with *E. coli* GyrA, nor was it able to bind to *E. coli* GyrA on an affinity column. We also used a yeast two-hybrid assay to demonstrate that AtGyrB3 does not bind to AtGyrA, or form a dimer, in yeast cells. The observation that AtGyrB1 but not AtGyrB2 binds to AtGyrA is intriguing (Table 2), given that AtGyrB2 was found to catalyse supercoiling in the presence of *E. coli* GyrA [9]. It is feasible that AtGyrA is not the partner subunit of AtGyrB2; this is a subject for further investigation.

AtGyrB3 has previously been found to complement the *E. coli gyrB^ts^* strain N4177 [9]. This experiment had been performed using *GYRB3* in plasmid pET17b, in which expression is controlled by the bacteriophage T7 promoter; complementation relied on leaky expression from this promoter. We repeated this experiment using *GYRB3* in the plasmid pKER15 with the gene under the control of the *E. coli gyrB* promoter. *GYRB3* did not complement the *gyrB^ts^* mutation at the non-permissive temperature.

It therefore appears that AtGyrB3 is not a GyrB subunit. Analysis of microarray data revealed that *GYRB3* expression is highest in the stamens and pollen. A yeast two-hybrid screen revealed several proteins that may interact with AtGyrB3; microarray data revealed that some of these also appear to be primarily expressed in pollen. It may be that AtGyrB3 has a role in meiosis in pollen nuclei, as it has putative nuclear localisation signals and a DNA-binding domain. Lack of obvious meiotic phenotypes in *GYRB3* mutants might be due to genetic redundancies.

## Materials and Methods

### Bioinformatic analyses

Targeting was predicted using MultiLoc [12]; protein sequence alignments were carried out using the Match-Box Server [13]; structure predictions were carried out using SWISS-MODEL [20] and the Eukaryotic Linear Motif server [21]. Expression analysis was carried out using the GENEVESTIGATOR database [26].

### Plant material, growth conditions and genotypic analysis

Three independent T-DNA insertion alleles (SAIL_61_B05, SAIL_390_D05, SALK_108979) were obtained from the Nottingham Arabidopsis Stock Centre (NASC). Plants were grown on plates containing Murashige and Skoog salts, pH 5.8, 1% (w/v) sucrose, and 0.5% (w/v) phytagel under continuous light at 25°C. The following gene-specific oligonucleotides were used for genotypic analysis in combination with the left border-specific oligonucleotides SI (5′-TTC ATA ACC AAT CTC GAT ACA C-3′) for SAIL lines and SK (5′-TGG TTC ACG TAG TGG GCC ATC G-3′) for the SALK line: 61F (5′-TAA CAA TAT TTT CTC CAA GTC GG-3′) and 61R (5′-CCC TGA CAC CAA AGA TGA TGC-3′) for SAIL_61_B05 and SALK_108979; 390F (5′-CCC TCT TGA CAG CCA AAT CAG-3′) and 390R (5′-GAG AAG TCG AAG CTT GTT GAG G-3′) for SAIL_390_D05.

### RACE PCR analysis

5′ RACE reactions were performed using the 2^nd^ Generation 5′ RACE kit (Roche), following the manufacturer's instructions. The primers used were: 5′-TTA CAG GCT AAC GGC TTC TA-3′ and 5′-GCG CAA TGT TTA TGG TGG TAC C-3′.

For 3′ RACE, the GeneRacer RACE Ready cDNA kit (Invitrogen) was used according to the manufacturer's instructions. Three consecutive nested PCRs were performed to obtain specific 3′ fragment using the following AtGyrB3 gene-specific oligonucleotides: GSP1 5′-GAA CTG GTG TTT GGC CAA CAT ATA G-3′, GYRB3F1 5′-CTA AAG AAG ACA GTT CAC TC-3′, GYRB3Fnes 5′-TTC TCC GAG GTC AAC CAA TTG C-3′. The product was cloned into pGEM-T (Promega) and sequenced.

### Complementation of temperature sensitive *E. coli*

The *E. coli* temperature sensitive strain N4177 [33] was made electrically competent [34], and transformed with plasmids by electroporation (Eppendorf Electroporator 2510), with the expression lag at 30°C. Following transformation, the cells were selected by growth on ampicillin (100 µg/mL). To test complementation, single colonies of the transformants were streaked onto LB agar plates containing ampicillin, which were incubated at 30°C and 43°C overnight.

### Cloning, expression and protein purification

*AtGYRB3* was cloned into the expression vectors pET17b and pET19b (Promega); expression was tried in various *E. coli* strains, with inductions at different temperatures. AtGyrB3 protein was produced from pET17b in Rosetta pLysS cells (Stratagene) after incubation at 37°C for 4 h. The cells were harvested by centrifugation, and the pellets were resuspended in 1 M Tris·HCl (pH 8.8) containing 10% sucrose, and quick-frozen in liquid nitrogen, and stored at −80°C until use. The resuspended pellets were thawed, and transferred to Snakeskin dialysis tubing (Pierce) with a molecular weight cut off of 10 kDa. The protein was then dialysed for 3.5 h at 7°C in 50 mM Tris·HCl (pH 8.0), 100 mM KCl, 2 mM DTT, 1 mM EDTA, 10% w/v glycerol, 8 M urea. A staged dialysis into buffer containing no urea was then performed; the soluble and insoluble proteins were analysed by SDS-PAGE. The soluble AtGyrB3 produced by refolding denatured protein was purified by Heparin Sepharose (Amersham) chromatography using an NaCl gradient. Fractions were quick-frozen in liquid nitrogen and stored at −80°C. Purified protein was shown to be AtGyrB3 by MALDI-TOF analysis.

### Supercoiling assays

DNA supercoiling assays were performed in 30 µl volumes [35]. Gyrase subunits (from either *E. coli* or *Arabidopsis*, prepared a described previously [9], [30]) at concentrations up to 200 nM, were incubated with 0.5 µg of relaxed pBR322 DNA, under the following reaction conditions: 35 mM Tris·HCl, 24 mM KCl, 4.4 mM DTT, 4 mM MgCl_2_, 1.8 mM spermidine, 0.36 mg/ml acetylated BSA, 9 µg/ml tRNA, 6.5% (w/v) glycerol and 1.4 mM ATP at pH 7.5. Reactions were incubated at 37°C for 1 h, and were terminated by the addition of equal volumes of chloroform:isoamyl alcohol (24∶1) and 40% sucrose, 100 mM Tris·HCl (pH 8), 100 mM EDTA, 0.5 µg/ml Bromophenol Blue. Samples were analysed by agarose gel electrophoresis (1%).

### GyrA-affinity column

A GyrA-affinity column was made as previously described [30]; the column was first re-equilibrated with five column volumes of 50 mM Tris·HCl (pH 8.0), 1 mM EDTA, 1 mM DTT, 10% glycerol. *E. coli* GyrB and AtGyrB3 were applied to the column and eluted with the same buffer containing either 0.5 M or 2 M NaCl. The fractions were analysed by SDS-PAGE.

### Yeast two-hybrid analysis

Yeast two-hybrid analysis was performed using the Matchmaker LexA Two-Hybrid System (Clontech), according to the manufacturer's instructions.

## Supporting Information

Figure S1 Schematic representation of the AtGYRB3 gene structure and the positions of the T-DNA insertions. The T-DNA of line SAIL_61_B05 and SALK_108979 are inserted in the 5′ UTR of locus A5g04110, whereas line SAIL_390_D05 harbors the T-DNA insertion in the fourth exon. Red arrows represent the position of the left border.
(0.78 MB DOC)

Figure S2 Sequence alignments. A: Alignment of the full-length amino acid sequences (including transit peptides) of *E. coli* GyrB, AtGyrB1, AtGyrB2 and AtGyrB3. Identical residues are shaded dark red. Similar residues are shaded light red. Conserved motifs found in all type II topoisomerases are bordered in black. B: Alignment of the transducer sequences of the GyrB proteins from *E. coli*, *Mycobacterium tuberculosis*, *A. thaliana*, *Nicotiana benthamiana*, and *Oryza sativa*. Identical aligned amino acids are shaded dark red. Similar amino acids are shaded light red. The amino acids bordered in black correspond to Lys337 in *E. coli* GyrB. C: Alignment of the predicted AtGyrB3 SANT domain with the SANT domains of *Saccharomyces cerevisiae* SWI3, *S. cerevisiae* ADA2, mouse N-CoR and *S. cerevisiae* TFIIIB B′. The three conserved aromatic amino acids are bordered in black.
(0.66 MB DOC)
