# Supplementary material for: Arabidopsis thaliana GYRB3 Does Not Encode a DNA Gyrase Subunit
Source: PLoS One. 2010 Mar 26;5(3):e9899. doi: 10.1371/journal.pone.0009899 (PMC2845627; doi:10.1371/journal.pone.0009899)
Supplement: Figure S1 — Schematic representation of the AtGYRB3 gene structure and the positions of the T-DNA insertions. The T-DNA of line SAIL_61_B05 and SALK_108979 are inserted in the 5′ UTR of locus A5g04110, whereas line SAIL_390_D05 harbors the T-DNA insertion in the fourth exon. Red arrows represent the position of the left border. (0.78 MB DOC) [file pone.0009899.s001.doc]

Fig. S1


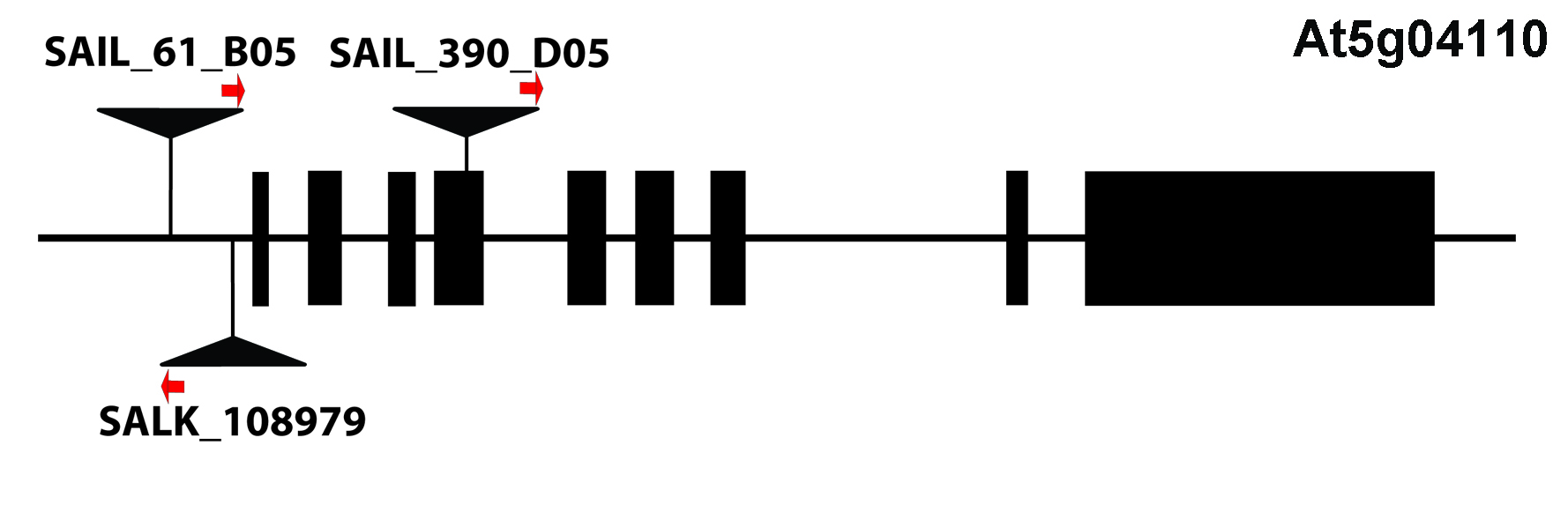


Fig. S1. Schematic representation of the AtGYRB3 gene structure and the positions of the T-DNA insertions. The T-DNA of line SAIL_61_B05 and SALK_108979 are inserted in the 5' UTR of locus A5g04110, whereas line SAIL_390_D05 harbors the T-DNA insertion in the fourth exon. Red arrows represent the position of the left border.
