# Supplementary material for: Arabidopsis thaliana GYRB3 Does Not Encode a DNA Gyrase Subunit
Source: PLoS One. 2010 Mar 26;5(3):e9899. doi: 10.1371/journal.pone.0009899 (PMC2845627; doi:10.1371/journal.pone.0009899)
Supplement: Figure S2 — Sequence alignments. A: Alignment of the full-length amino acid sequences (including transit peptides) of E. coli GyrB, AtGyrB1, AtGyrB2 and AtGyrB3. Identical residues are shaded dark red. Similar residues are shaded light red. Conserved motifs found in all type II topoisomerases are bordered in black. B: Alignment of the transducer sequences of the GyrB proteins from E. coli, Mycobacterium tuberculosis, A. thaliana, Nicotiana benthamiana, and Oryza sativa. Identical aligned amino acids are shaded dark red. Similar amino acids are shaded light red. The amino acids bordered in black correspond to Lys337 in E. coli GyrB. C: Alignment of the predicted AtGyrB3 SANT domain with the SANT domains of Saccharomyces cerevisiae SWI3, S. cerevisiae ADA2, mouse N-CoR and S. cerevisiae TFIIIB B′. The three conserved aromatic amino acids are bordered in black. (0.66 MB DOC) [file pone.0009899.s002.doc]

| EcGYRB  A | - | - | - | - | - | - | - | - | - | - | - | - | - | - | - | - | - | - | - | - | - | - | - | - | - | - | - | - | - | - | - | - | - | - | - | - | - | - | - | - | - | - | - | - | - | - | - | - | - | - |  | 0 |
| --- | --- | --- | --- | --- | --- | --- | --- | --- | --- | --- | --- | --- | --- | --- | --- | --- | --- | --- | --- | --- | --- | --- | --- | --- | --- | --- | --- | --- | --- | --- | --- | --- | --- | --- | --- | --- | --- | --- | --- | --- | --- | --- | --- | --- | --- | --- | --- | --- | --- | --- | --- | --- |
| AtGYRB1 | M | A | L | V | Q | R | Q | H | S | Y | L | L | R | Y | F | R | L | M | A | S | R | P | R | P | R | L | F | S | H | S | L | Y | P | S | L | H | R | H | S | S | A | L | S | S | - | - | - | S | T | P |  | 47 |
| AtGYRB2 | M | A | L | L | Q | R | A | S | Y | L | R | L | Y | Y | L | R | L | M | G | S | R | P | - | - | R | L | F | S | S | S | L | S | P | A | L | H | R | H | S | S | T | L | S | S | P | P | F | S | S | P |  | 48 |
| AtGYRB3 | - | - | - | - | - | - | - | - | - | - | - | - | - | - | - | - | - | - | - | - | - | - | - | - | - | - | - | - | - | - | - | - | - | - | - | - | - | - | - | - | - | - | - | - | - | - | - | - | - | - |  | 0 |
|  |  |  |  |  |  |  |  |  |  |  |  |  |  |  |  |  |  |  |  |  |  |  |  |  |  |  |  |  |  |  |  |  |  |  |  |  |  |  |  |  |  |  |  |  |  |  |  |  |  |  |  |  |
| EcGYRB | - | - | - | - | - | - | - | - | - | - | - | - | - | - | - | - | - | - | - | - | - | - | - | - | - | - | - | - | - | - | - | - | - | - | - | - | - | - | - | - | - | M | S | N | S | Y | D | S | S | S |  | 9 |
| AtGYRB1 | - | - | - | - | R | I | K | F | Q | L | A | N | V | F | S | Q | R | L | V | Q | R | N | A | V | S | P | K | S | F | M | S | S | T | M | E | S | L | Q | E | S | S | T | S | K | D | Y | S | S | E | H |  | 93 |
| AtGYRB2 | S | P | S | F | R | L | K | F | Q | L | T | S | V | L | S | Q | R | L | I | Q | R | N | A | I | S | S | R | - | F | L | S | T | - | - | E | A | S | Q | E | T | T | T | S | K | G | Y | S | S | E | Q |  | 95 |
| AtGYRB3 | - | - | - | - | - | - | - | - | - | - | - | - | - | - | - | - | - | - | - | - | - | - | - | - | - | - | - | - | - | - | - | - | - | - | - | - | - | - | - | - | - | - | - | - | - | - | - | - | - | - |  | 0 |
|  |  |  |  |  |  |  |  |  |  |  |  |  |  |  |  |  |  |  |  |  |  |  |  |  |  |  |  |  |  |  |  |  |  |  |  |  |  |  |  |  |  |  |  |  |  |  |  |  |  |  |  |  |
| EcGYRB | I | K | V | L | K | G | L | D | A | V | R | K | R | P | G | M | Y | I | G | D | T | D | D | G | T | G | L | H | H | M | V | F | E | V | V | D | N | A | I | D | E | A | L | A | G | H | C | K | E | I |  | 59 |
| AtGYRB1 | I | Q | V | L | E | G | L | D | P | V | R | K | R | P | G | M | Y | I | G | S | T | G | S | - | R | G | L | H | H | L | V | Y | E | I | L | D | N | A | I | D | E | A | Q | A | G | F | A | S | K | I |  | 142 |
| AtGYRB2 | I | Q | V | L | E | G | L | D | P | V | R | K | R | P | G | M | Y | I | G | S | T | G | S | - | R | G | L | H | H | L | V | Y | E | I | L | D | N | A | I | D | E | A | Q | A | G | Y | A | S | K | V |  | 144 |
| AtGYRB3 | - | - | - | - | - | - | - | - | - | - | - | - | - | - | - | - | - | - | - | - | - | - | - | - | - | - | - | - | - | - | - | - | - | - | - | - | - | - | - | - | - | - | - | - | - | - | - | - | - | - |  | 0 |
|  |  |  |  |  |  |  |  |  |  |  |  |  |  |  |  |  |  |  |  |  |  |  |  |  |  |  |  |  |  |  |  |  |  |  |  |  |  |  |  |  |  |  |  |  |  |  |  |  |  |  |  |  |
| EcGYRB | I | V | T | I | H | A | D | N | S | V | S | V | Q | D | D | G | R | G | I | P | T | G | I | H | P | E | E | G | V | S | A | A | E | V | I | M | T | V | L | H | A | G | G | K | F | D | D | N | S | - |  | 108 |
| AtGYRB1 | D | V | V | L | H | S | D | D | S | V | S | I | S | D | N | G | R | G | I | P | T | D | L | H | P | A | T | G | K | S | S | L | E | T | V | L | T | V | L | H | A | G | G | K | F | G | G | K | S | S |  | 192 |
| AtGYRB2 | D | V | V | L | H | A | D | G | S | V | S | V | V | D | N | G | R | G | I | P | T | D | L | H | P | A | T | K | K | S | S | L | E | T | V | L | T | V | L | H | A | G | G | K | F | G | G | T | S | S |  | 194 |
| AtGYRB3 | - | - | - | - | - | - | - | - | - | - | - | - | - | - | - | - | - | - | - | - | - | - | - | - | - | - | - | - | - | - | - | - | - | - | - | - | - | - | M | Y | S | G | - | - | - | - | - | - | - | - |  | 4 |
|  |  |  |  |  |  |  |  |  |  |  |  |  |  |  |  |  |  |  |  |  |  |  |  |  |  |  |  |  |  |  |  |  |  |  |  |  |  |  |  |  |  |  |  |  |  |  |  |  |  |  |  |  |
| EcGYRB | - | Y | K | V | S | G | G | L | H | G | V | G | V | S | V | V | N | A | L | S | Q | K | L | E | L | V | I | Q | R | E | G | K | I | H | R | Q | I | Y | E | H | G | V | P | Q | A | P | L | A | V | T |  | 157 |
| AtGYRB1 | G | Y | S | V | S | G | G | L | H | G | V | G | L | S | V | V | N | A | L | S | E | A | L | E | V | I | V | R | R | D | G | M | E | F | Q | Q | K | Y | S | R | G | K | P | V | T | T | L | T | C | H |  | 242 |
| AtGYRB2 | G | Y | S | V | S | G | G | L | H | G | V | G | L | S | V | V | N | A | L | S | E | A | L | E | V | S | V | W | R | D | G | M | E | H | K | Q | N | Y | S | R | G | K | P | I | T | T | L | T | C | R |  | 244 |
| AtGYRB3 | - | - | - | - | - | - | - | - | - | - | - | - | - | - | T | V | K | R | V | S | P | L | Q | D | I | K | L | R | - | - | - | - | - | - | - | - | - | - | - | - | - | - | - | - | - | - | - | - | - | - |  | 18 |
|  |  |  |  |  |  |  |  |  |  |  |  |  |  |  |  |  |  |  |  |  |  |  |  |  |  |  |  |  |  |  |  |  |  |  |  |  |  |  |  |  |  |  |  |  |  |  |  |  |  |  |  |  |
| EcGYRB | G | E | T | E | K | T | - | - | - | - | G | T | M | V | R | F | W | P | S | L | E | T | - | - | - | F | T | N | V | T | E | F | E | Y | E | I | L | A | K | R | L | R | E | L | S | F | L | N | S | G |  | 200 |
| AtGYRB1 | V | L | P | P | E | S | R | G | T | Q | G | T | C | I | R | F | W | P | D | K | E | G | F | A | L | F | T | T | A | I | Q | F | D | H | N | T | I | A | G | R | I | R | E | L | A | F | L | N | P | K |  | 292 |
| AtGYRB2 | V | L | P | L | E | S | K | G | T | K | G | T | S | I | R | F | W | P | D | K | E | - | - | - | V | F | T | T | A | I | E | F | D | H | N | T | I | A | G | R | I | R | E | L | A | F | L | N | P | K |  | 291 |
| AtGYRB3 | - | - | - | - | - | - | - | - | - | - | - | - | - | - | - | - | - | - | - | - | - | - | - | - | - | - | - | - | - | - | - | - | - | - | - | - | - | - | - | - | - | - | - | - | - | - | - | - | V | Q |  | 20 |
|  |  |  |  |  |  |  |  |  |  |  |  |  |  |  |  |  |  |  |  |  |  |  |  |  |  |  |  |  |  |  |  |  |  |  |  |  |  |  |  |  |  |  |  |  |  |  |  |  |  |  |  |  |
| EcGYRB | V | S | I | R | L | R | D | K | R | D | G | K | E | - | - | - | - | D | H | F | H | Y | E | G | G | I | K | A | F | V | E | Y | L | N | K | N | K | T | P | I | H | P | N | I | F | Y | F | S | T | E |  | 246 |
| AtGYRB1 | V | T | I | S | L | K | K | E | D | D | D | P | E | R | D | V | Y | S | E | Y | F | Y | A | G | G | L | T | E | Y | V | S | W | L | N | T | D | K | K | P | L | H | - | D | V | L | G | F | R | K | E |  | 341 |
| AtGYRB2 | V | T | I | S | L | K | K | E | D | D | D | P | E | K | T | Q | Y | S | E | Y | S | F | A | G | G | L | T | E | Y | V | S | W | L | N | T | D | K | N | P | I | H | - | D | V | L | G | F | R | R | E |  | 340 |
| AtGYRB3 | I | T | V | S | L | E | M | E | D | N | D | P | N | - | - | - | - | K | E | Y | L | Y | A | K | G | L | S | E | F | V | T | W | L | N | A | D | K | K | P | L | H | - | D | V | L | G | F | R | K | E |  | 65 |
|  |  |  |  |  |  |  |  |  |  |  |  |  |  |  |  |  |  |  |  |  |  |  |  |  |  |  |  |  |  |  |  |  |  |  |  |  |  |  |  |  |  |  |  |  |  |  |  |  |  |  |  |  |
| EcGYRB | K | D | G | I | G | V | E | V | A | L | Q | W | N | - | D | G | F | Q | E | N | I | Y | C | F | T | N | N | I | P | Q | R | D | G | G | T | H | L | A | G | F | R | A | A | M | T | R | T | L | N | A |  | 295 |
| AtGYRB1 | I | N | G | S | T | V | D | V | S | L | Q | W | C | S | D | A | Y | S | D | T | M | L | G | Y | A | N | S | I | R | T | I | D | G | G | T | H | I | E | G | V | K | A | S | L | T | R | T | L | N | S |  | 391 |
| AtGYRB2 | I | N | G | A | T | V | D | V | A | L | Q | W | C | S | D | A | Y | S | D | T | M | L | G | Y | A | N | S | I | R | T | I | D | G | G | T | H | I | E | G | V | K | A | S | L | T | R | T | L | N | T |  | 390 |
| AtGYRB3 | I | N | G | T | T | I | N | I | A | L | Q | W | C | V | D | G | Y | S | N | K | I | L | G | Y | A | N | G | I | R | T | M | D | G | G | T | Y | I | D | G | V | K | A | S | I | T | R | T | L | N | S |  | 115 |
|  |  |  |  |  |  |  |  |  |  |  |  |  |  |  |  |  |  |  |  |  |  |  |  |  |  |  |  |  |  |  |  |  |  |  |  |  |  |  |  |  |  |  |  |  |  |  |  |  |  |  |  |  |
| EcGYRB | Y | M | D | K | E | G | Y | S | K | K | A | K | V | S | A | T | G | D | D | A | R | E | G | L | I | A | V | V | S | V | K | V | P | D | P | K | F | S | S | Q | T | K | D | K | L | V | S | S | E | V |  | 345 |
| AtGYRB1 | L | A | K | K | L | K | V | I | K | E | K | D | I | S | L | S | G | E | H | V | R | E | G | L | T | C | I | V | S | V | K | V | P | N | P | E | F | E | G | Q | T | K | T | R | L | G | N | P | E | V |  | 441 |
| AtGYRB2 | L | A | K | K | S | K | T | V | K | E | K | D | I | S | L | S | G | E | H | V | R | E | G | L | T | C | I | V | S | V | K | V | P | N | P | E | F | E | G | Q | T | K | T | R | L | G | N | P | E | V |  | 440 |
| AtGYRB3 | L | V | E | K | S | K | L | V | E | D | K | D | I | I | F | T | E | E | H | V | M | E | G | L | T | C | I | V | S | V | I | V | P | K | P | E | F | E | G | Q | T | Q | R | - | L | G | N | P | N | V |  | 164 |
|  |  |  |  |  |  |  |  |  |  |  |  |  |  |  |  |  |  |  |  |  |  |  |  |  |  |  |  |  |  |  |  |  |  |  |  |  |  |  |  |  |  |  |  |  |  |  |  |  |  |  |  |  |
| EcGYRB | K | S | A | V | E | Q | Q | M | N | E | L | L | A | E | Y | L | L | E | N | P | T | D | A | K | I | V | V | G | K | I | I | D | A | A | R | A | R | E | A | A | R | R | A | R | E | M | T | R | R | K |  | 395 |
| AtGYRB1 | R | K | I | V | D | Q | S | V | Q | E | Y | L | T | E | Y | L | E | L | H | P | D | V | L | E | S | I | I | S | K | S | L | N | A | Y | K | A | A | L | A | A | K | R | A | R | E | L | V | R | S | K |  | 491 |
| AtGYRB2 | R | K | I | V | D | Q | S | V | Q | E | Y | L | T | E | F | L | E | L | H | P | D | I | L | E | S | I | I | S | K | S | L | N | A | Y | K | A | A | L | A | A | K | R | A | R | E | L | V | R | S | K |  | 490 |
| AtGYRB3 | R | E | I | V | D | Q | S | V | Q | E | C | L | M | E | S | F | E | L | H | P | D | V | F | E | S | I | M | S | K | S | Y | N | A | Y | K | T | D | L | A | V | K | R | A | R | D | V | Y | S | S | E |  | 214 |
|  |  |  |  |  |  |  |  |  |  |  |  |  |  |  |  |  |  |  |  |  |  |  |  |  |  |  |  |  |  |  |  |  |  |  |  |  |  |  |  |  |  |  |  |  |  |  |  |  |  |  |  |  |
| EcGYRB | G | A | L | D | L | A | G | L | P | G | K | L | A | D | C | Q | E | R | D | P | A | L | S | E | L | Y | L | V | E | G | D | S | A | G | G | S | A | K | Q | G | R | N | R | K | N | Q | - | - | - | - |  | 441 |
| AtGYRB1 | S | V | L | K | S | S | S | L | P | G | K | L | A | D | C | S | S | T | D | P | A | E | S | E | I | F | I | V | E | G | D | S | A | G | G | S | A | K | Q | G | R | D | R | R | F | Q | - | - | - | - |  | 537 |
| AtGYRB2 | S | V | L | K | S | S | S | L | P | G | K | L | A | D | C | S | S | T | D | P | E | V | S | E | I | F | I | V | E | G | D | S | A | G | G | S | A | K | Q | G | R | D | R | R | F | Q | - | - | - | - |  | 536 |
| AtGYRB3 | S | V | A | M | V | C | A | I | E | S | I | P | M | K | L | T | N | S | S | S | E | T | S | E | T | F | I | G | R | G | V | S | S | G | G | A | A | K | H | D | S | D | R | C | F | K | N | K | R | T |  | 264 |
|  |  |  |  |  |  |  |  |  |  |  |  |  |  |  |  |  |  |  |  |  |  |  |  |  |  |  |  |  |  |  |  |  |  |  |  |  |  |  |  |  |  |  |  |  |  |  |  |  |  |  |  |  |
| EcGYRB | - | - | - | - | - | - | - | - | A | I | L | P | L | K | G | - | K | I | L | N | V | E | K | A | R | F | D | K | M | L | S | S | Q | E | V | A | T | L | I | T | A | L | G | C | G | I | G | R | D | E |  | 482 |
| AtGYRB1 | - | - | - | - | - | - | - | - | A | I | L | P | L | R | G | - | K | I | L | N | I | E | R | K | D | E | A | A | M | Y | K | N | E | E | I | Q | N | L | I | L | G | L | G | L | G | V | K | G | E | D |  | 578 |
| AtGYRB2 | - | - | - | - | - | - | - | - | A | I | L | P | L | R | G | - | K | I | L | N | I | E | R | K | D | E | A | A | M | Y | K | N | E | E | I | Q | N | L | I | L | G | L | G | L | G | V | K | G | E | D |  | 577 |
| AtGYRB3 | W | E | Q | P | W | S | D | D | A | V | S | T | A | P | G | E | S | S | E | N | T | S | G | S | S | F | E | K | K | S | K | K | P | K | S | S | V | S | S | S | H | L | C | F | S | S | R | Q | S | A |  | 314 |
|  |  |  |  |  |  |  |  |  |  |  |  |  |  |  |  |  |  |  |  |  |  |  |  |  |  |  |  |  |  |  |  |  |  |  |  |  |  |  |  |  |  |  |  |  |  |  |  |  |  |  |  |  |
| EcGYRB | Y | N | P | D | K | L | R | Y | H | S | I | I | I | M | T | D | A | D | V | D | G | - | - | - | - | - | - | - | S | H | I | R | T | L | L | L | T | F | F | Y | R | Q | M | P | E | I | V | E | R | G |  | 525 |
| AtGYRB1 | F | N | K | E | N | L | R | Y | H | K | I | I | I | L | T | D | A | D | V | D | G | - | - | - | - | - | - | - | A | H | I | R | T | L | L | L | T | F | F | F | R | Y | Q | R | A | L | F | D | A | G |  | 621 |
| AtGYRB2 | F | K | K | E | N | L | R | Y | H | K | I | I | I | L | T | D | A | D | V | D | G | - | - | - | - | - | - | - | A | H | I | R | T | L | L | L | T | F | F | F | R | Y | Q | R | A | L | F | D | A | G |  | 620 |
| AtGYRB3 | D | D | A | N | S | P | S | P | K | D | V | S | N | K | T | P | K | D | V | T | H | G | S | N | K | D | V | S | N | K | T | S | K | D | V | I | T | H | G | S | N | K | T | R | P | A | I | P | I | G |  | 364 |
|  |  |  |  |  |  |  |  |  |  |  |  |  |  |  |  |  |  |  |  |  |  |  |  |  |  |  |  |  |  |  |  |  |  |  |  |  |  |  |  |  |  |  |  |  |  |  |  |  |  |  |  |  |
| EcGYRB | H | V | Y | I | A | Q | P | P | L | Y | K | V | K | K | G | K | Q | E | Q | Y | I | K | D | D | E | A | M | D | Q | Y | Q | I | S | I | A | L | D | G | A | T | L | H | T | N | A | S | A | P | A | L |  | 575 |
| AtGYRB1 | C | I | Y | V | G | V | P | P | L | F | K | V | E | R | G | K | Q | A | H | Y | C | Y | D | D | - | - | - | - | - | - | - | - | - | - | - | - | - | - | - | - | - | - | - | - | - | - | - | - | - | - |  | 645 |
| AtGYRB2 | C | I | Y | V | G | V | P | P | L | F | K | V | E | R | G | K | N | A | Q | Y | C | Y | D | D | - | - | - | - | - | - | - | - | - | - | - | - | - | - | - | - | - | - | - | - | - | - | - | - | - | - |  | 644 |
| AtGYRB3 | P | R | F | Q | A | E | I | P | V | W | I | A | P | T | K | K | G | K | F | Y | G | S | P | G | D | S | N | T | L | R | W | L | G | T | G | - | - | - | - | - | - | - | - | - | - | - | - | - | V | W |  | 401 |
|  |  |  |  |  |  |  |  |  |  |  |  |  |  |  |  |  |  |  |  |  |  |  |  |  |  |  |  |  |  |  |  |  |  |  |  |  |  |  |  |  |  |  |  |  |  |  |  |  |  |  |  |  |
| EcGYRB | A | G | E | A | L | E | K | L | V | S | E | Y | N | A | T | Q | K | M | I | N | R | M | E | R | R | Y | P | K | A | M | L | K | E | L | I | Y | Q | P | T | L | T | E | A | D | L | S | D | E | Q | T |  | 625 |
| AtGYRB1 | - | - | A | A | L | K | K | I | T | A | S | F | P | G | N | - | - | - | - | - | - | - | - | - | - | - | - | - | - | - | - | - | - | - | - | - | - | - | - | - | - | - | - | - | - | - | - | - | - | - |  | 658 |
| AtGYRB2 | - | - | A | D | L | K | K | I | T | S | N | F | P | A | N | - | - | - | - | - | - | - | - | - | - | - | - | - | - | - | - | - | - | - | - | - | - | - | - | - | - | - | - | - | - | - | - | - | - | - |  | 657 |
| AtGYRB3 | P | T | Y | S | L | K | K | T | V | H | S | K | K | V | G | E | G | R | S | D | S | C | S | C | A | S | P | R | S | T | N | - | - | - | - | - | - | - | - | - | - | - | - | - | - | - | - | - | - | - |  | 432 |
|  |  |  |  |  |  |  |  |  |  |  |  |  |  |  |  |  |  |  |  |  |  |  |  |  |  |  |  |  |  |  |  |  |  |  |  |  |  |  |  |  |  |  |  |  |  |  |  |  |  |  |  |  |
| EcGYRB | V | T | R | W | V | N | A | L | V | S | E | L | N | D | K | E | Q | H | G | S | Q | W | K | F | D | V | H | T | N | A | E | Q | N | L | F | E | P | I | V | R | V | R | T | H | G | V | D | T | D | Y |  | 675 |
| AtGYRB1 | - | - | - | - | - | - | - | - | - | - | - | - | - | - | - | - | - | - | - | - | - | - | - | - | - | - | - | - | - | - | - | - | - | - | - | - | - | - | - | - | - | - | - | - | - | - | - | - | - | - |  | 658 |
| AtGYRB2 | - | - | - | - | - | - | - | - | - | - | - | - | - | - | - | - | - | - | - | - | - | - | - | - | - | - | - | - | - | - | - | - | - | - | - | - | - | - | - | - | - | - | - | - | - | - | - | - | - | - |  | 657 |
| AtGYRB3 | - | - | - | - | - | - | - | - | - | - | - | - | - | - | - | - | - | - | - | - | - | - | - | - | - | - | - | - | - | - | - | - | - | - | - | - | - | - | - | - | - | - | - | - | - | - | - | - | - | - |  | 432 |
|  |  |  |  |  |  |  |  |  |  |  |  |  |  |  |  |  |  |  |  |  |  |  |  |  |  |  |  |  |  |  |  |  |  |  |  |  |  |  |  |  |  |  |  |  |  |  |  |  |  |  |  |  |
| EcGYRB | P | L | D | H | E | F | I | T | G | G | E | Y | R | R | I | C | T | L | G | E | K | L | R | G | L | L | E | E | D | A | F | I | E | R | G | E | R | R | Q | P | V | A | S | F | E | Q | A | L | D | W |  | 725 |
| AtGYRB1 | - | - | - | - | - | - | - | - | - | - | - | - | - | - | - | - | - | - | - | - | - | - | - | - | - | - | - | - | - | - | - | - | - | - | - | - | - | - | - | - | - | A | S | Y | N | - | - | - | - | - |  | 662 |
| AtGYRB2 | - | - | - | - | - | - | - | - | - | - | - | - | - | - | - | - | - | - | - | - | - | - | - | - | - | - | - | - | - | - | - | - | - | - | - | - | - | - | - | - | - | A | S | Y | N | - | - | - | - | - |  | 661 |
| AtGYRB3 | - | - | - | - | - | - | - | - | - | - | - | - | - | - | - | - | - | - | C | I | K | R | H | K | K | E | A | Q | E | L | L | E | K | E | I | N | R | A | F | S | T | W | E | F | D | Q | M | G | E | E |  | 464 |
|  |  |  |  |  |  |  |  |  |  |  |  |  |  |  |  |  |  |  |  |  |  |  |  |  |  |  |  |  |  |  |  |  |  |  |  |  |  |  |  |  |  |  |  |  |  |  |  |  |  |  |  |  |
| EcGYRB | L | V | K | E | S | R | R | G | L | S | I | Q | R | Y | K | G | L | G | E | M | N | P | E | Q | L | W | E | T | T | M | D | P | E | S | R | R | M | L | R | V | T | V | K | D | A | I | A | A | D | Q |  | 775 |
| AtGYRB1 | - | - | - | - | - | - | - | - | - | - | I | Q | R | F | K | G | L | G | E | M | M | P | A | Q | L | W | E | T | T | M | N | P | D | T | R | I | L | K | Q | L | V | V | D | D | A | A | E | T | N | M |  | 702 |
| AtGYRB2 | - | - | - | - | - | - | - | - | - | - | I | Q | R | F | K | G | L | G | E | M | M | P | E | Q | L | W | E | T | T | M | N | P | E | T | R | I | L | K | Q | L | V | V | D | D | I | A | E | A | N | M |  | 701 |
| AtGYRB3 | I | V | L | K | S | W | T | A | K | E | E | R | R | F | E | A | L | V | K | K | N | P | L | S | S | S | D | G | F | W | E | F | A | S | N | A | F | P | Q | K | S | K | K | D | L | L | S | Y | Y | Y |  | 514 |
|  |  |  |  |  |  |  |  |  |  |  |  |  |  |  |  |  |  |  |  |  |  |  |  |  |  |  |  |  |  |  |  |  |  |  |  |  |  |  |  |  |  |  |  |  |  |  |  |  |  |  |  |  |
| EcGYRB | L | F | T | T | L | M | G | D | A | V | E | P | R | R | A | F | I | E | E | N | A | L | K | A | - | - | A | N | I | D | I | - |  |  |  |  |  |  |  |  |  |  |  |  |  |  |  |  |  |  |  | 804 |
| AtGYRB1 | V | F | S | S | L | M | G | A | R | V | D | V | R | K | E | L | I | K | S | A | A | T | R | M | N | L | E | N | L | D | I | - |  |  |  |  |  |  |  |  |  |  |  |  |  |  |  |  |  |  |  | 733 |
| AtGYRB2 | T | F | S | S | L | M | G | A | R | V | D | V | R | K | E | L | I | K | N | A | A | T | R | I | N | L | Q | R | L | D | I | - |  |  |  |  |  |  |  |  |  |  |  |  |  |  |  |  |  |  |  | 732 |
| AtGYRB3 | N | V | F | L | I | K | R | M | R | L | L | K | S | S | A | A | N | N | I | D | S | D | D | D | H | Y | D | D | F | L | A | G |  |  |  |  |  |  |  |  |  |  |  |  |  |  |  |  |  |  |  | 546 |

B


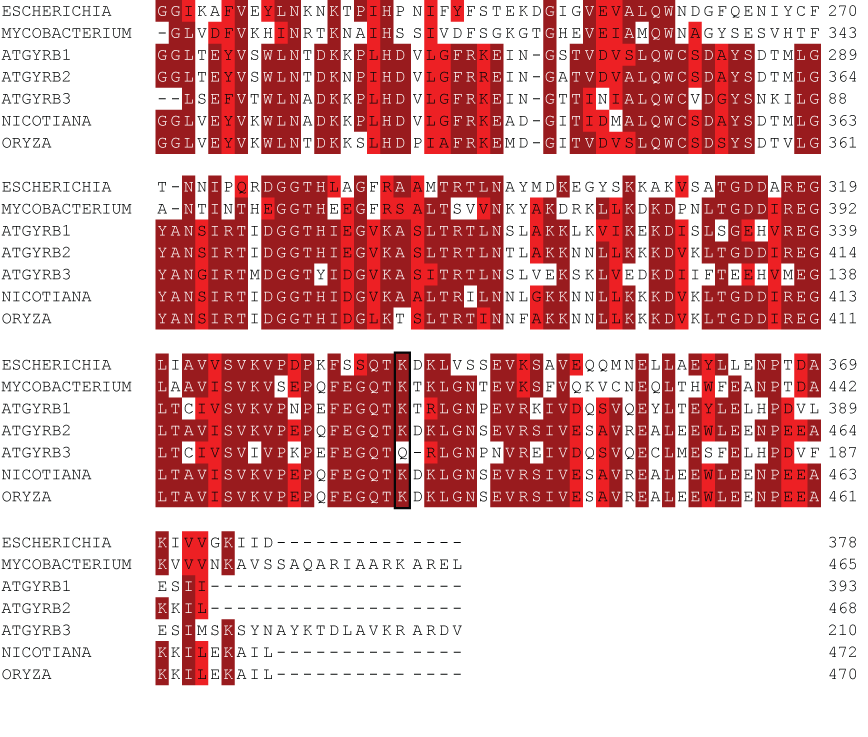


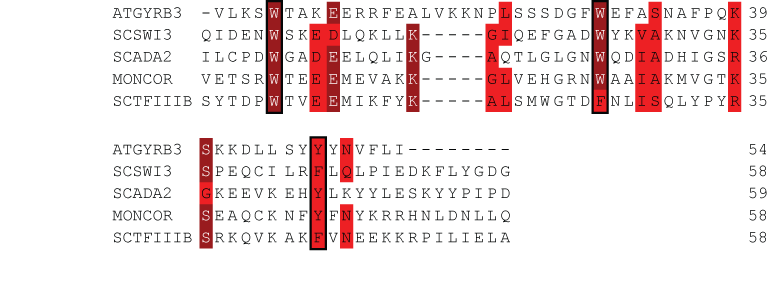


C

**Figure S2. Sequence alignments.** A: Alignment of the full-length amino acid sequences (including transit peptides) of *E. coli* GyrB, AtGyrB1, AtGyrB2 and AtGyrB3. Identical residues are shaded dark red. Similar residues are shaded light red. Conserved motifs found in all type II topoisomerases are bordered in black. B: Alignment of the transducer sequences of the GyrB proteins from *E. coli*, *Mycobacterium tuberculosis*, *A. thaliana*, *Nicotiana benthamiana*, and *Oryza sativa*. Identical aligned amino acids are shaded dark red. Similar amino acids are shaded light red. The amino acids bordered in black correspond to Lys337 in *E. coli* GyrB. C: Alignment of the predicted AtGyrB3 SANT domain with the SANT domains of *Saccharomyces cerevisiae* SWI3, *S. cerevisiae* ADA2, mouse N-CoR and *S. cerevisiae* TFIIIB B''. The three conserved aromatic amino acids are bordered in black.
